# Bone marrow mesenchymal stem cellsderived exosomes stabilize atherosclerosis through inhibiting pyroptosis

**DOI:** 10.1186/s12872-023-03453-y

**Published:** 2023-09-07

**Authors:** Zhibin Bai, Haolin Hu, Fangfang Hu, Jiajie Ji, Zhenling Ji

**Affiliations:** 1 Center of Interventional Radiology and Vascular Surgery, Department of Radiology, Medical School, Zhongda Hospital, Southeast University, 87 Dingjiaqiao Road, Nanjing, 210009 Jiangsu China; 2 Department of General Surgery, Institute for Minimally Invasive Surgery, Medical School, ZhongDa Hospital, Southeast University, 87 Dingjiaqiao Road, Nanjing, 210009 Jiangsu China

**Keywords:** Atherosclerosis, Bone marrow mesenchymal stem cells, Exosomes, Pyroptosis

## Abstract

**Objectives:**

This study aimed to determine the effects of bone marrow mesenchymal stem cells (BMSCs)-derived exosomes (BMSC-EXO) on atherosclerosis (AS), and its related underlying mechanisms.

**Methods:**

Exosomes were isolated from mouse BMSCs, and identified by transmission electron microscopy (TEM), Nanosight (NTA), and western blot. A mouse AS model was established, and exosomes were injected into the tail vein. Total cholesterol (TC) and triglycerides (TG) were detected using their corresponding assay kits. The contents of IL-1β and IL-18 in serum were detected by ELISA. The mRNA and protein expression levels of GSDMD, Caspase1, and NLRP3 were detected by qRT-PCR and Western blot. Finally, aortic tissues in the Model and BMSC-EXO groups were sent for sequencing.

**Results:**

TEM, NTA, and western blot indicated successful isolation of exosomes. Compared with the control group, the TC, TG contents, IL-1β and IL-18 concentrations of the mice in the Model group were significantly increased; nonetheless, were significantly lower after injected with BMSC-EXO than those in the Model group (p < 0.05). Compared with the control group, the expressions of NLRP3, caspase-1 and GSDMD were significantly up-regulated in the Model group (p < 0.05), while the expressions of NLRP3, caspase-1, and GSDMD were significantly down-regulated by BMSC-EXO. By sequencing, a total of 3852 DEGs were identified between the Model and BMSC-EXO group and were significantly enriched in various biological processes and pathways related to mitochondrial function, metabolism, inflammation, and immune response.

**Conclusion:**

AS can induce pyroptosis, and BMSC-EXO can reduce inflammation and alleviate the progression of AS by inhibiting NLRP3/Caspase-1/GSDMD in the pyroptosis pathway.

**Supplementary Information:**

The online version contains supplementary material available at 10.1186/s12872-023-03453-y.

## Introduction

Atherosclerosis (AS) is a chronic inflammatory disease, triggered by endothelial dysfunction and structural changes, involving chronic inflammation of the vessel wall [1, 2]. Studies have shown that the formation of AS is closely related to a unique inflammation-related programmed cell death cell called pyroptosis [3]. Pyroptosis is a new form of cell death in which the inflammasome causes the cleavage and multimerization of the inflammatory Caspase substrate GSDMD protein, resulting in cell disintegration and the release of pro-inflammatory cytokines such as IL-1β and IL-18 [4, 5]. It is reported that AS-related risk factors (oxidized LDL and cholesterol crystals) can trigger NLRP3 inflammasome activation and Caspase-1 production in macrophages or endothelial cells. Caspase-1-mediated pyroptosis with inflammatory cytokines (IL-1β and IL-18) could promote the formation and development of AS [6, 7]. Trimethylamine N-oxide was also found to promote ApoE−/− mice AS by triggering vascular endothelial cell pyroptosis via the NLRP3/caspase-1/GSDMD pathway [8]. Bai et al. showed that miR-302c-3p exerted an anti-pyroptosis effect in vivo and in vitro by directly targeting NLRP3 and inhibiting NLRP3 inflammasome activation [9]. Therefore, inhibition of pyroptosis can be a new target for the prevention and treatment of AS.

Bone marrow mesenchymal stem cells (BMSCs) have a high degree of self-replication ability and multi-directional differentiation potential. BMSCs can differentiate into bone and cartilage tissues, muscle and muscle tissues, muscle cells, nerve cells, etc. under different induction conditions [10]. There are increasing evidences that BMSCs can effectively treat severe diseases, including traumatic brain injury, acute respiratory distress syndrome, and acute kidney injury [11–13]. It has been reported that the efficacy of BMSCs may be attributed to the paracrine secretion of trophic factors, anti-inflammatory proteins, and exosomes [14]. Exosomes are lipid bilayer membrane vesicles, about 50 to 150 nm in diameter, produced by various cells including BMSCs, cancer cells, etc. [15, 16]. Exosomes are key regulators of intercellular communication by transporting cargoes (proteins, lipids, DNA and RNA) from protocells to recipient cells. Recent studies have shown that BMSCs-derived exosomes (BMSC-EXO) have positive roles in AS treatment [17, 18]. Lin et al. showed that exosomal miR-125b-5p from BMSCs could suppress atherosclerotic plaque formation via inhibiting Map4k4 expression [17]. Another study reported that BMSCs-microvesicles harboring miR-223 inhibited NLRP3 expression, and reduced macrophage pyroptosis to stabilize atherosclerotic plaques [18]. However, the pyroptosis and other molecular mechanisms of BMSC-EXO in AS remain unclear.

In this study, we constructed an AS mouse model and then injected BMSC-EXO into the tail vein of AS mice to study the effect of BMSC-EXO on AS by regulating cell pyroptosis. Subsequently, the aortas of mice in the model group and the BMSC-EXO-injected group were sequenced to explore the potential molecular mechanism of BMSC-EXO in the treatment of AS.

## Materials and methods

### Ethics statement

The study got the approval of the Clinical Ethical Committee of ZhongDa Hospital, Medical School, Southeast University.

### Isolation and identification of BMSC-EXOs

BMSCs were purchased from Procell Life Science & Technology Co., Ltd. (CP-M131, Wuhan, China). Cells were cultured in α-MEM complete medium containing 10% fetal bovine serum and 1% penicillin-streptomycin dual antibody. When the density of BMSCs reaches 80-90%, the medium was discarded, and the cells were washed with PBS three times. Then, exosome-free serum medium was added. After cultured for 48 h, the cell supernatant was collected, and transferred to a 50 mL centrifuge tube for gradient centrifugation at 4 °C (500×g, 5 min; 2000×g, 30 min). Subsequently, the supernatant was centrifuged at 120,000×g for 70 min at 4 °C, the supernatant was discarded, and the centrifugation operation was repeated once again. Exosomes were suspended in PBS and frozen at -80 ℃ for later use.

The concentrations of the isolated exosomes were determined using a BCA protein assay kit (Thermo Fisher Scientific, MA, USA) following the manufacturer’s instructions. After that, a NanoSight nanoparticle size analyzer (NS300, Malvern) was used to determine the exosome particle size. Exosome morphology was observed using transmission electron microscopy (JEM-1230, Joel) [19]. Exosome-specific proteins CD9, CD63 and Alix were detected using western blot with their corresponding antibodies (1:500) [20].

### Animals

Eight 4-6-week-old female ApoE-/- knockout mice and four 4-6-week-old female C57BL/6 mice were purchased from Changzhou Cavens Laboratory Animal Co., Ltd (Changzhou, China). Mice were routinely reared for 1 week in a constant temperature environment at 25 °C to acclimate to the environment. Starting from the second week, ApoE-/- mice were fed a high-fat diet (10% sucrose, 10% lard, 2% cholesterol and 0.5% bile acids), and C57BL/6 mice were fed conventional feeding.

### Establishment and grouping of animal models

After 4 weeks of feeding, ApoE-/- model mice (n = 4, BMSC-EXO group) were given tail vein injection of BMSC-EXO (400 μg/kg/W) [21] for 8 consecutive weeks as the BMSC-EXO group. Another 4 ApoE-/- model mice served as Model group, and 4 C57BL/6 mice with normal diet served as control group.

At the 12th week, whole blood of mice in three groups was collected, centrifuged at 1900 g for 10 min at 4 °C to separate serum and stored at -80 °C for subsequent detection of total cholesterol (TC), triglyceride (TG) and enzyme-linked immunosorbent assay (ELISA). After the mice were sacrificed, aortic tissue was harvested, and partially embedded in paraffin for Hematoxylin and Eosin (HE) Staining. The other part was stored at -80 °C for RNA extraction and real-time PCR (RT-PCR), western blot detection and Transcriptome sequencing.

### Hematoxylin and eosin (HE) staining and Masson staining

Aortic tissues of different groups were fixed in 4% paraformaldehyde, and then dehydrated with decreasing ethanol gradient (100, 95, 80 and 70%). After paraffin embedding, the aortic tissue samples were cut into 4 μm thick sections. After deparaffinized, the sections were stained with hematoxylin (BASO, BA-4097, China) for 6 min, and eosin (BASO, BA-4099) for 90 s. After dehydration, the sections were mounted with neutral gum. Masson staining was performed on slices using Masson (Servicebio, G1006, China) according to the relevant instructions provided in the manual. The sections were dehydrated, transparent, mounted, and then viewed with a microscope and photographed.

### Oil Red O staining

Lipid accumulation of the aorta was determined by oil red O staining (Servicebio, G1015). Aorta was fixed with 4% paraformaldehyde. Then, the aorta was stained with 0.5% oil red O solution (Servicebio, G1015, Wuhan, China) for 1 h, and then differentiated in a 60% propylene glycol solution. Lastly, the sections were viewed under a microscope.

### TdT-mediated dUTP-biotin nick end-labeling (TUNEL) staining

TUNEL staining was performed using the TUNEL Kit (G1507, Servicebio, China). Briefly, paraffin sections were dewaxed and hydrated, and repaired with proteinase K (G1205, Servicebio, China). The sections were then incubated with Triton X-100 (G1204 Servicebio, China), followed by 3% hydrogen peroxide solution. After that, the sections were reacted with TUNEL reaction solution and Streptavidin-HRP solution in turn. The sections were then subjected to DAB color development and hematoxylin staining. Finally, the sections were dehydrated, made transparent, mounted, and viewed under a microscope.

### TC and TG examinations

TC and TG contents in the mouse serum was examined using total cholesterol detection kit (Nanjing Jiancheng Bioengineering Institute, China) and triglyceride detection kit (Nanjing Jiancheng Bioengineering Institute, China), respectively, according to the instructions from the manufacturer.

### ELISA

The level of IL-1β and IL-18 in the mouse serum was detected using their corresponding ELISA detection kit (ELK Biotechnology) following the manufacturer’s protocols. Then, optical density (OD) values were measured at a wavelength of 450 nm using a microplate reader (MK3, Thermo).

### RT-PCR

The total RNA of aortic tissues was extracted using RNAiso plus kit (Takara Biotechnology, Dalian, China) following the manufacturer’s manual. Then, the total RNA was reverse transcribed into cDNA using the PrimeScript RTreagent kit (Perfect Real Time, Takara, Japan). Subsequently, real-time PCR was performed using a real-time PCR machine (LongGeen), and the cycling program included an initial denaturation step for 10 min at 95 °C, followed by a total of 40 cycles at 95 °C for 15 s, and at 60 °C for 60 s). All primers were synthesized by Shanghai Sangon Bioengineering Technology Service Co., Ltd., and the sequences of all primers were shown in Table 1. The mRNA expression of *GSDMD*, *Caspase1*, *NLRP3*, *Ucp1*, *Acsl5*, *Plin1*, *Acsl1*, *Fabp1*, *Scd4*, *Apoa1* and *Pltp* were calculated using the 2^−ΔΔCt^ method.

**Table 1:** The sequences of all primers

Primer	Sequence (5′–3′)
GAPDH-mF	GGTGAAGGTCGGTGTGAACG
GAPDH-mR	CTCGCTCCTGGAAGATGGTG
CASPASE-1-mF	ACAAGGCACGGGACCTATG
CASPASE-1-mR	TCCCAGTCAGTCCTGGAAATG
NLRP3-mF	ATTACCCGCCCGAGAAAGG
NLRP3-mR	TCGCAGCAAAGATCCACACAG
GSDMD-mF	CCATCGGCCTTTGAGAAAGTG
GSDMD-mR	ACACATGAATAACGGGGTTTCC
Ucp1-mF	GTGAACCCGACAACTTCCGAA
Ucp1-mR	TGCCAGGCAAGCTGAAACTC
Acsl5-mF	TCCTGACGTTTGGAACGGC
Acsl5-mR	CTCCCTCAATCCCCACAGAC
Plin1-mF	CAAGCACCTCTGACAAGGTTC
Plin1-mR	GTTGGCGGCATATTCTGCTG
Acsl1-mF	TGCCAGAGCTGATTGACATTC
Acsl1-mR	GGCATACCAGAAGGTGGTGAG
Fabp1-mF	ATGAACTTCTCCGGCAAGTACC
Fabp1-mR	GGTCCTCGGGCAGACCTAT
Scd4-mF	GCCCACTTGCCACAAGAGAT
Scd4-mR	GTAGCTGGGGTCATACAGATCA
Apoa1-mF	GGCACGTATGGCAGCAAGAT
Apoa1-mR	CCAAGGAGGAGGATTCAAACTG
Pltp-mF	CGCAAAGGGCCACTTTTACTA
Pltp-mR	GCCCCCATCATATAAGAACCAG

### Western blot

Total protein was extracted from aortic tissues using RIPA lysis buffer (P0013B, Beyotime), and the concentrations of the isolated total protein were measured using a BCA protein assay kit (PL212989, Thermo). The collected protein samples were separated by 10% SDS-PAGE, and then transferred to PVDF membranes (IPVH00010, Millipore). After blocked with 5% nonfat milk (0.75 g milk powder + 15 mL PBS) at 37 °C for 1 h, the membranes were incubated with anti-Caspase1 antibody (1:1000, ProteinTech, USA), anti-GSDMD antibody (1:1000, ProteinTech, USA), anti-NLRP3 antibody (1:1000, Abcam, USA) and anti-GAPDH rabbit polyclonal antibody (1:1000, ProteinTech, USA) overnight at 4 °C. After that, the membranes were incubated with the secondary antibody (Goat anti-rabbit IgG (H + L) -HRP, 1:5000, Jackson ImmunoResearch, USA) for 2 h. Protein bands were visualized by enhanced chemiluminescence.

### Transcriptome sequencing

The aortic tissues in the BMSC-EXO and Model group were sequenced by Yanzai Biotechnology (Shanghai) Co., Ltd. (Shanghai, China). Total RNA was extracted from both groups of tissues using RNAiso Plus (TaKaRa, Tokyo, Japan) and used for transcriptome sequencing as described previously [22]. Differential analysis of gene expression in BMSC-EXO and Model groups was performed using DESeq. The |log_2_Fold Change (FC)| > 1 and P-value < 0.05 were used as thresholds for screening differentially expressed genes (DEGs). Then, Gene Ontology (GO) and Kyoto Encyclopedia of Genes and Genomes (KEGG) enrichment analysis [23–25] was performed on these DEGs. In addition, 8 DEGs (4 up-regulated and 4 down-regulated) were selected for RT-qPCR validation, and the sequences of the 8 DEGs are shown in Table 1.

### Statistical analysis

All statistical graphs were drawn using Graphpad prism 8 (Graphpad Software, San Diego, CA). The mean ± standard deviation (SD) was used to express all the data. Comparisons between two groups were performed using Student’s t test, and for comparisons among more than two groups, one-way analysis of variance (ANOVA) was used. P < 0.05 was the screening criterion for significant difference.

## Results

### Characterization of BMSC-EXO

**Fig. 1:**
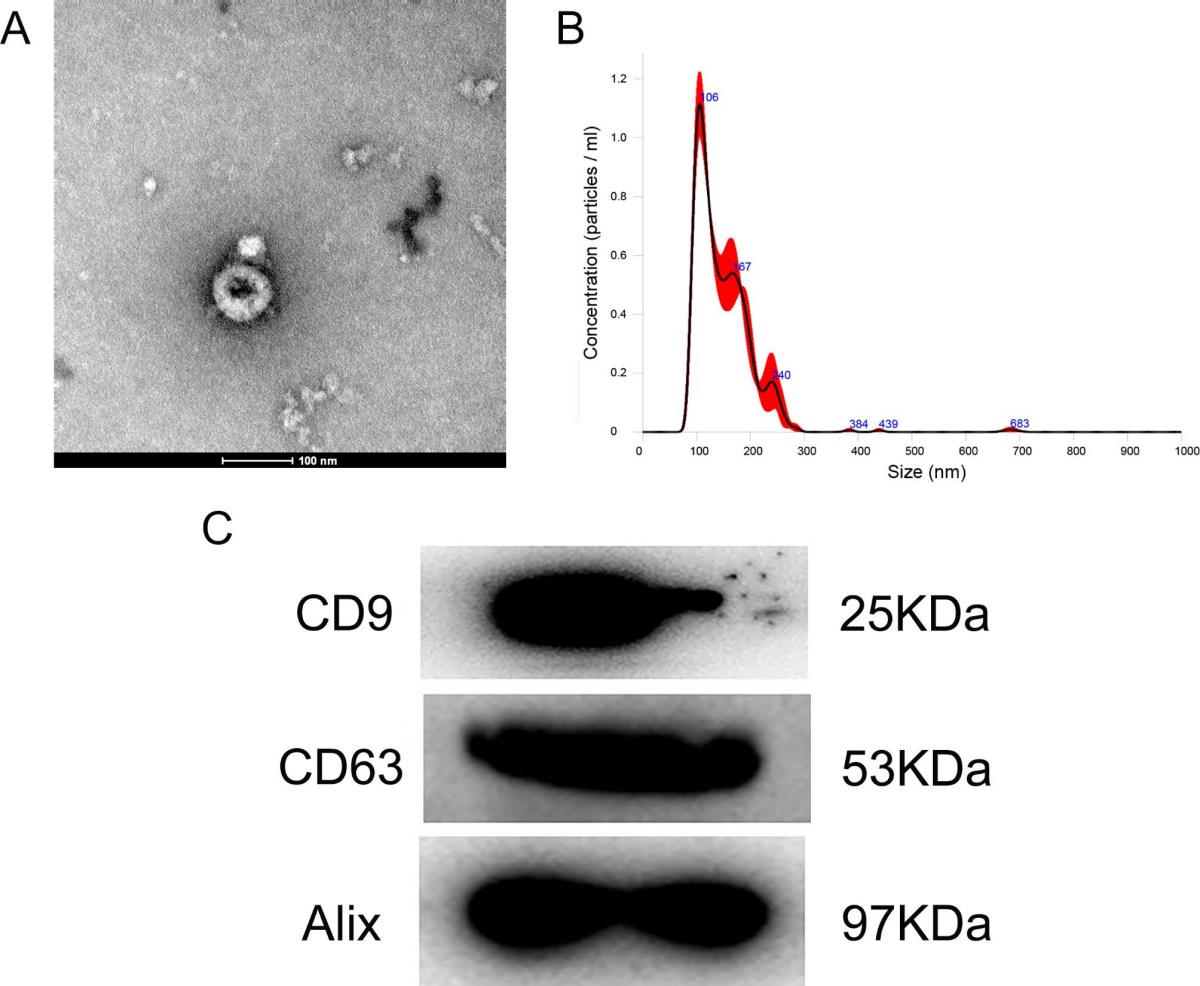
Characterization of mouse bone marrow mesenchymal stem cells (BMSCs)-derived exosomes (BMSC-EXO). (**A**) Transmission electron microscopy image of exosomes isolated from mouse BMSCs. (**B**) The size distribution of exosomes determined by nanoparticle tracking analysis. (**C**) The images of exosome-specific CD9, CD63, and Alix proteins examined by western blot

The exosomes isolated from mouse BMSCs were characterized using TEM, NTA, and western blot. TEM results showed that the isolated substances had a typical bilayer membrane and cup holder-like structure (Fig. 1A). The particle size was found to be approximately 148.3 nm by NTA (Fig. 1B). Western blot showed that exosome marker proteins CD9, CD63 and Alix were all expressed in exosomes (Fig. 1C). These results indicated that exosomes were successfully isolated from the supernatant of mouse BMSCs.

### BMSC-EXO improved the formation of atherosclerotic vulnerable plaques in ApoE (-/-) mice

**Fig. 2:**
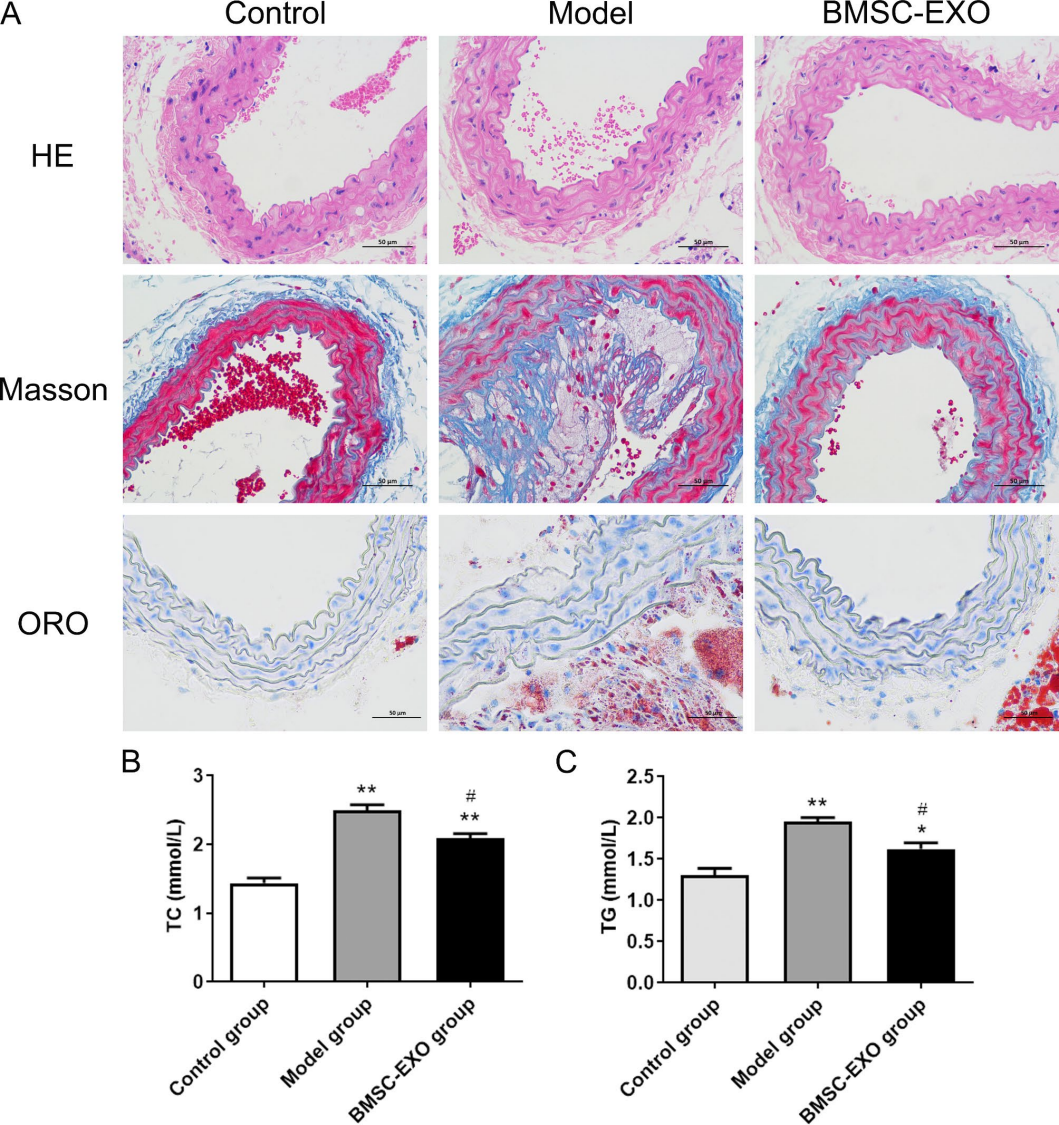
BMSC-EXO alleviates AS. (**A**) The histopathological changes in each group by HE staining, Masson staining and Oil Red O (ORO) staining. (**B**) Total cholesterol (TC) contents were measured using a TC detection kit. (**C**) Triglyceride (TG) contents were measured using a TG detection kit. *P < 0.05 vs. control group; **P < 0.01 vs. control group; ^#^*P* < 0.05 vs. Model group

A series of histological analyses were performed to examine the effect of injection of BMSC-EXO on AS-related pathology. HE staining showed that compared with the control group, the intima of the aorta in the model group presented ring-shaped diffuse thickening and formed typical atherosclerotic plaques. After injection of BMSC-EXO, the lesion area of the aortic root plaque was lower than that of the model group (Fig. 2A). In addition, Masson and Oil Red O staining showed that in the model group, there were a large number of willow-shaped cholesterol crystals in the plaque, thinner fibrous cap, and larger lipid pool (Fig. 2A). Compared with the model group, injection of BMSC-EXO significantly reduced the lipid area, increased the collagen content, and thickened the fibrous cap. Moreover, compared with the control group, the TC and TG contents of the mice in the Model group were significantly increased (p < 0.01, Fig. 2B and C). However, the contents of TC and TG in ApoE−/− mice injected with BMSC-EXO were significantly lower than those in the Model group (p < 0.05). These data indicate that BMSC-EXO may stabilize atherosclerotic plaques in vivo.

### BMSC-EXO inhibits pyroptosis through the NLRP3/caspase-1/GSDMD pathway

**Fig. 3:**
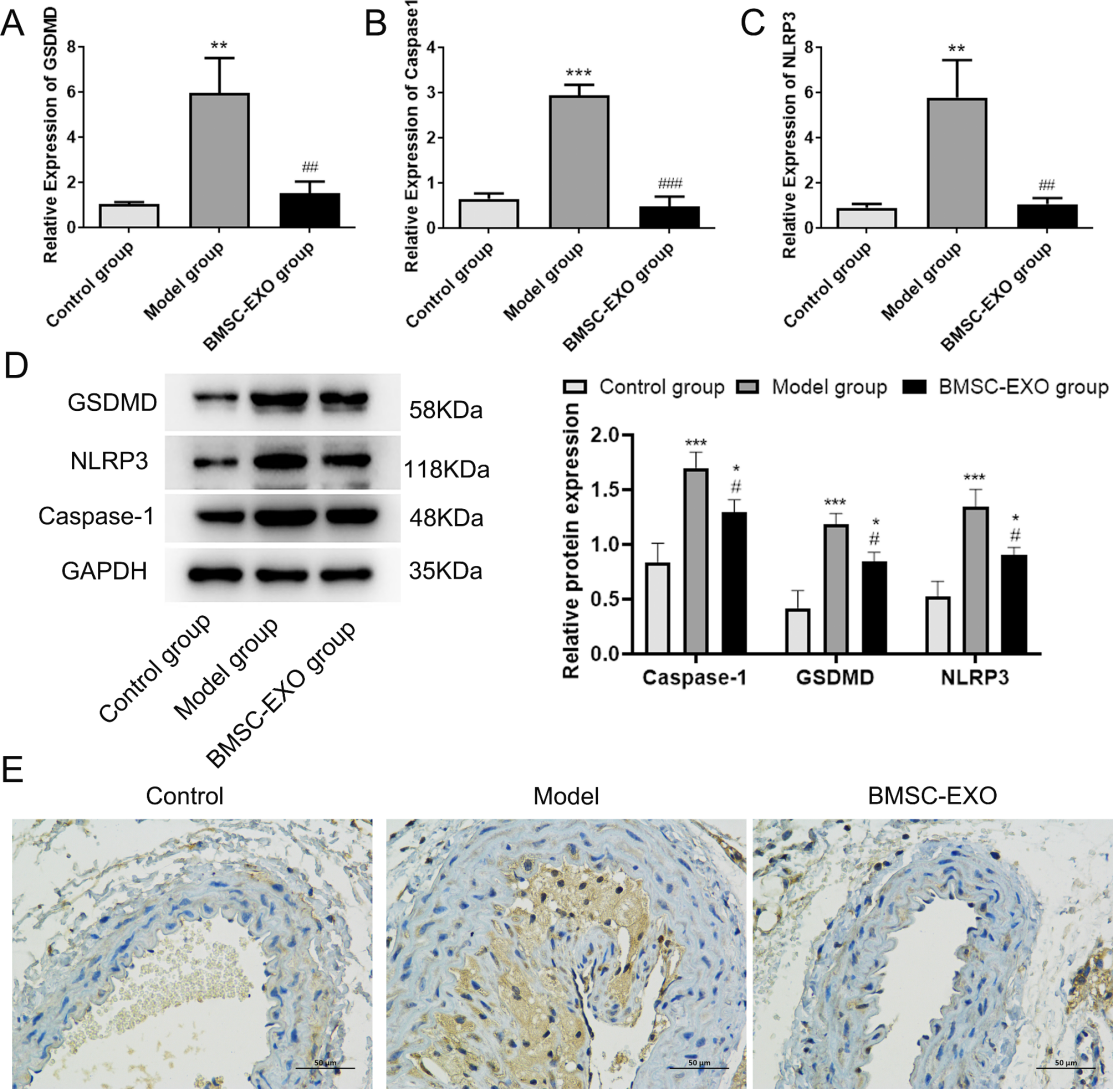
BMSC-EXO inhibited pyroptosis. The expression of GSDMD (**A**), Caspase1 (**B**) and NLRP3 (**C**) were measured by RT-qPCR. (**D**) The protein expressions of GSDMD, Caspase1 and NLRP3 determined by western blot. (**E**) Representative results of GSDMD immunohistochemical staining in each group. *P < 0.05 vs. control group; **P < 0.01 vs. control group; ***P < 0.001 vs. control group; ^#^*P* < 0.05 vs. Model group. ^##^*P* < 0.01 vs. Model group. ^###^*P* < 0.01 vs. Model group

**Fig. 4:**
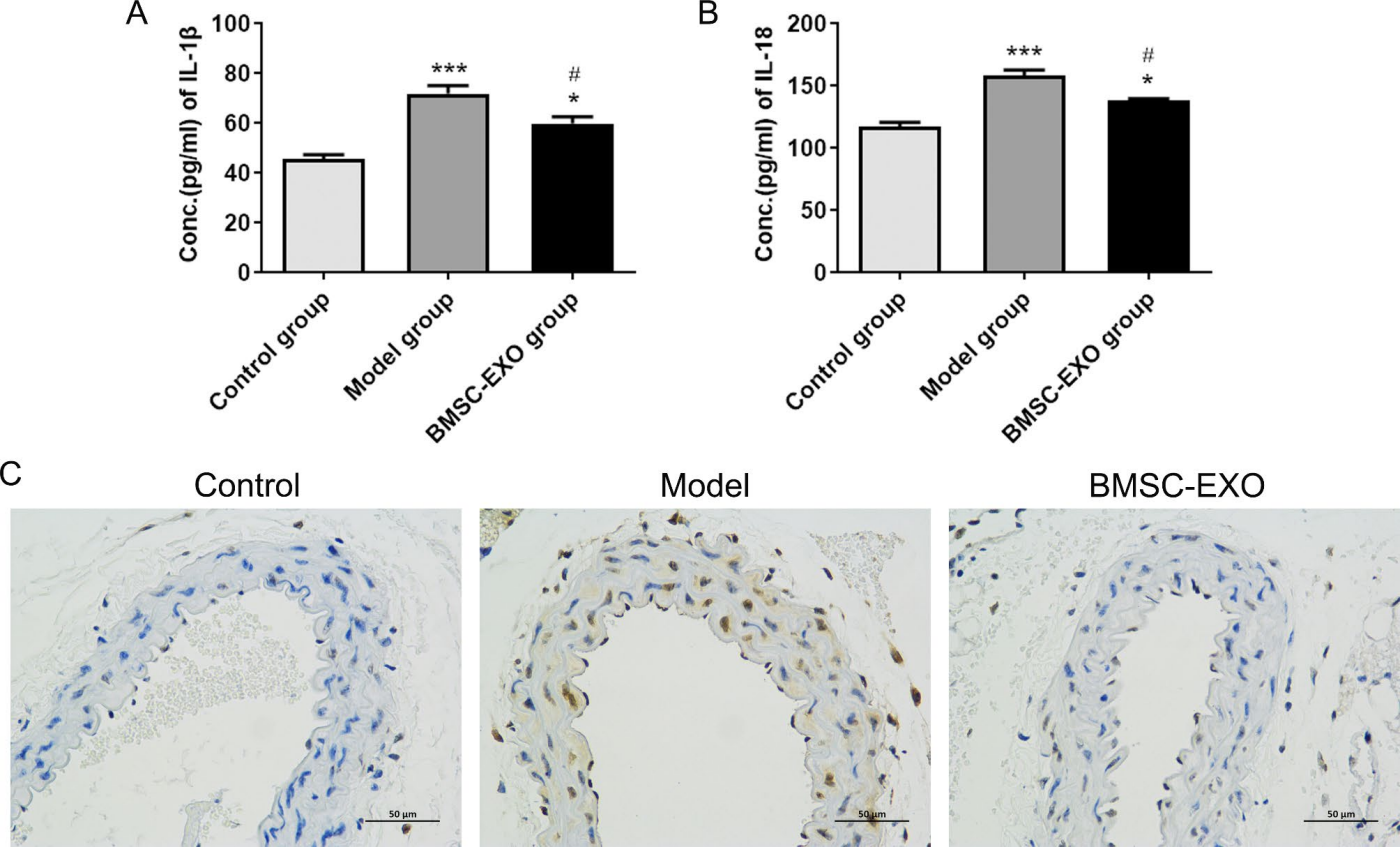
(**A**) Levels of IL-1β in mouse arterial tissues measured using ELISA. (**B**) Levels of IL-18 in mouse arterial tissues measured using ELISA. (**C**) Representative **r**esults of Tunel staining in each group. *P < 0.05 vs. control group; ***P < 0.001 vs. control group; ^#^*P* < 0.05 vs. Model group

Pyroptosis is a type of programmed cell death characterized by caspase-1 maturation, which eventually leads to the cleavage of the pore-forming protein GSDMD and the release of IL-1 and IL-18 [26]. qRT-PCR and western blot analysis showed that the expressions of *NLRP3*, *caspase-1* and *GSDMD* were significantly up-regulated in ApoE−/− mice compared with the control group (p < 0.05, Fig. 3A, B, C and D). However, ApoE−/− mice injected with BMSC-EXO exhibited down-regulated *NLRP3*, *caspase-1* and *GSDMD* compared with the Model group. Immunohistochemical results showed that the expression of GSDMD was enhanced in the model group, while the expression of GSDMD was significantly attenuated after BMSC-EXO injection (Fig. 3E). Furthermore, ELISA results showed that ApoE−/− mice had increased concentrations of IL-1β and IL-18, whereas the concentrations of IL-1β and IL-18 were decreased in ApoE-/- mice injected with BMSC-EXO (p < 0.05, Fig. 4A and B). TUNEL staining revealed a significant reduction of DNA fragments (TUNEL positive cells) in BMSC-EXO group compared with Model group (Fig. 4C). This suggested that BMSC-EXO could significantly inhibit the pyroptosis of AS cells.

### Identification of DEGs in BMSC-EXO-treated mice and functional analysis of these DEGs

**Fig. 5:**
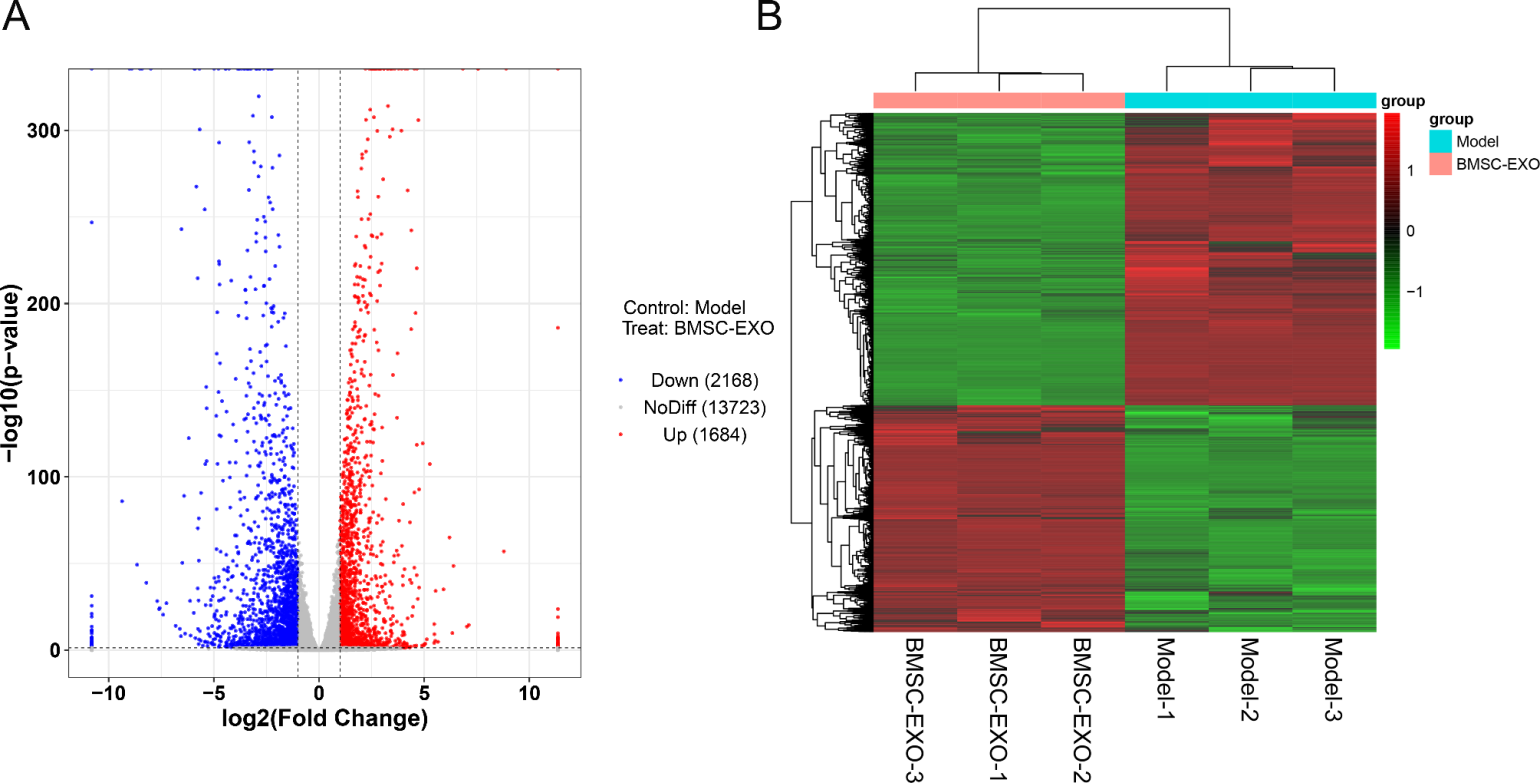
Expression profiles of differentially expressed genes (DEGs) between Model and BMSC-EXO group. (**A**) The volcano plot of DEGs. (**B**) The heat map of DEGs.

The aortic tissues of BMSC-EXO and Model group were sequenced, and the DEGs between BMSC-EXO and Model group were analyzed. Based on |log_2_FC| > 1 and P < 0.05, a total of 3852 DEGs were identified in BMSC-EXO group, including 1684 up-regulated DEGs and 2168 down-regulated DEGs (Fig. 5A). Then, the 3852 DEGs were used for bidirectional hierarchical cluster, and the results showed that these DEGs could well distinguish the Model and BMSC-EXO group ((Fig. 5B).

**Fig. 6:**
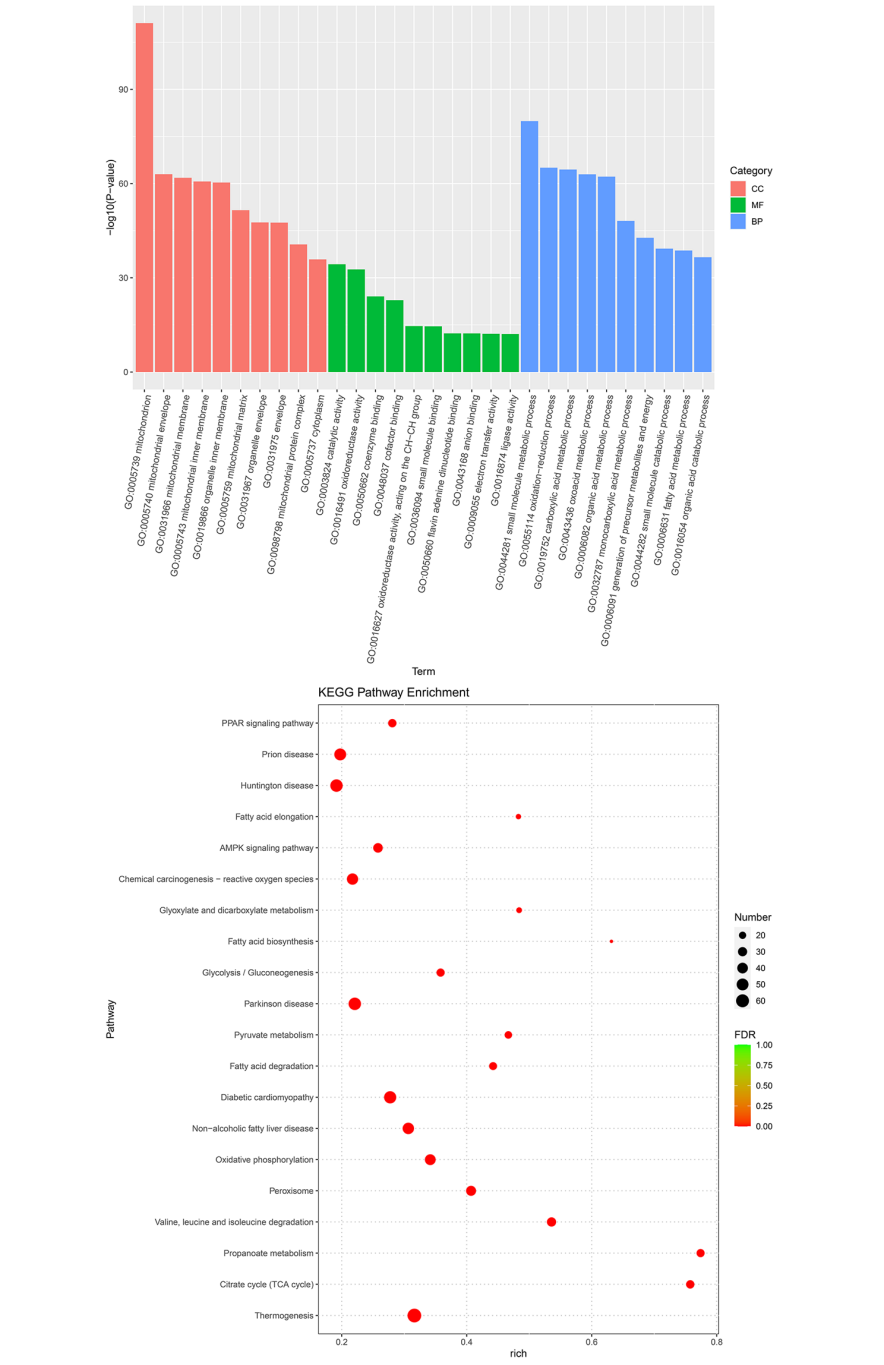
Gene Ontology (GO) terms and Kyoto Encyclopedia of Genes and Genomes (KEGG) pathways (www.keggip/kegg/kegg1.htmlanehisaaboratores.yoto.Japan) enrichment of up-regulated DEGs.

**Fig. 7:**
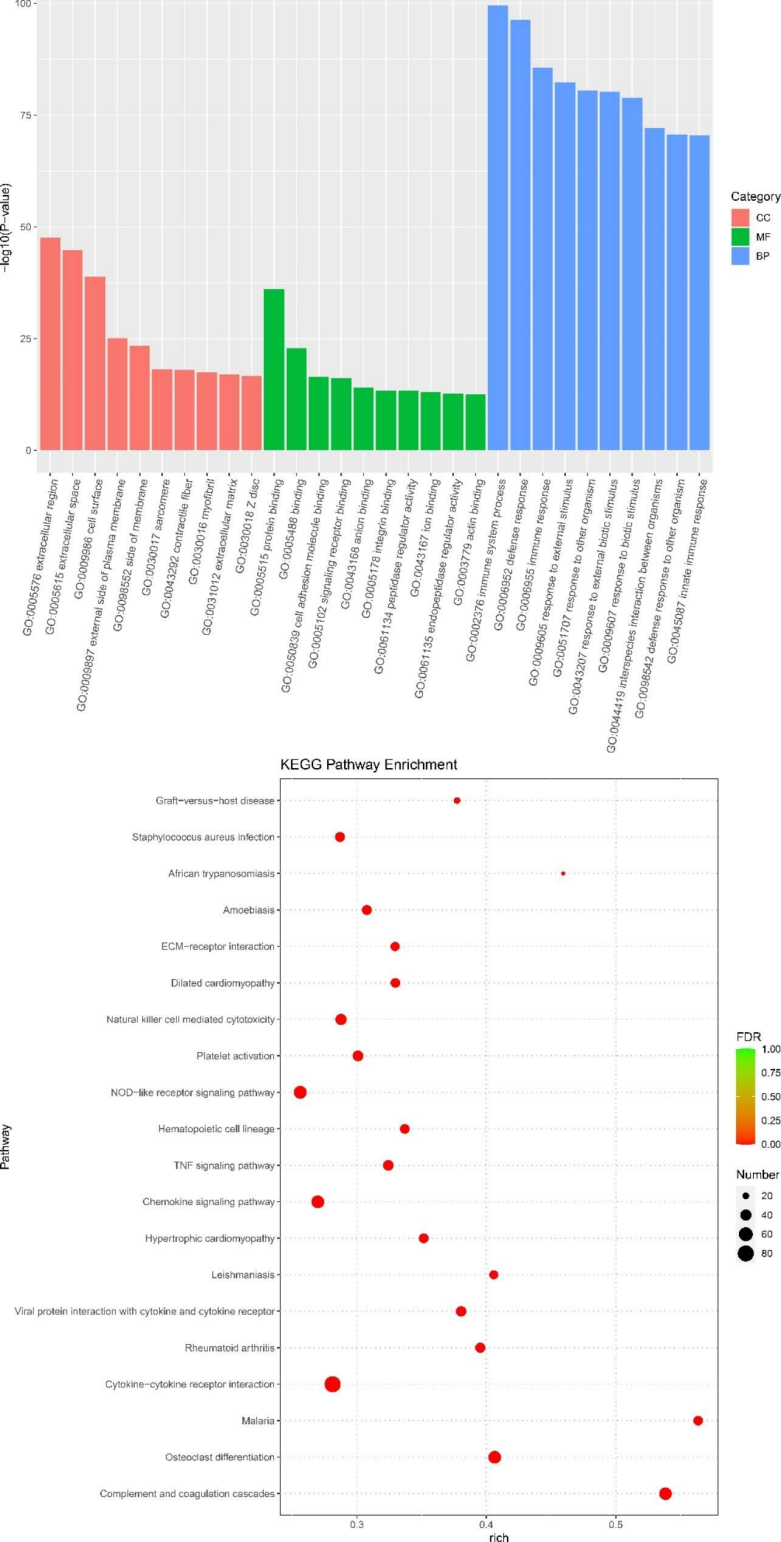
Gene Ontology (GO) terms and Kyoto Encyclopedia of Genes and Genomes (KEGG) pathways (www.keggip/kegg/kegg1.htmlanehisaaboratores.yoto.Japan) enrichment of down-regulated DEGs.

After that, these DEGs were submitted for functional analyses. Figures 6 and 7 shows the top 10 GO terms in cellular component (CC), molecular function (MF), and biological process (BP). It was found that these up-regulated DEGs were associated with the “mitochondrion” in CC, and “catalytic activity” and “oxidoreductase activity” in MF, “small molecule metabolic process”, “oxidation-reduction process”, and “carboxylic acid metabolic process” in BP, while these down-regulated DEGs were associated with the “extracellular region”, “extracellular space”, and “cell surface” in CC, and “binding” in MF, “immune system process”, “defense response”, and “immune response” in BP. Besides, these up-regulated DEGs were enriched in KEGG pathways, such as “Peroxisome”, “Thermogenesis”, “citrate cycle (TCA cycle)”, “propanoate metabolism”, “cytokine-cytokine receptor interaction” and “PPAR signaling pathway” (Fig. 6B). In contrast, the down-regulated DEGs were mainly enriched in were enriched in KEGG pathways, such as “Complement and coagulation cascades”, “Malaria”, “Cytokine-cytokine receptor interaction”, “Chemokine signaling pathway” (Fig. 7).

### RT-qPCR verification

We chose the eight DEGs for RT-qPCR verification, including 4 up-regulated DEGs (*Ucp1*, *Acsl5*, *Plin1* and *Acsl1*) and 4 down-regulated DEGs (*Fabp1*, *Scd4*, *Apoa1* and *Pltp*). Compared with the Model group, the expressions of *Ucp1*, *Acsl5* and *Acsl1* were significantly up-regulated in the BMSC-EXO group, while the expressions of *Scd4*, *Apoa1* and *Pltp* were significantly down-regulated in the BMSC-EXO group (P < 0.05, Figures S1A–F). However, the expression of *Plin1* and *Fabp1* was not significantly different between the two groups (P > 0.05, Figure S1G, H). The results showed that the concordance rate between sequencing analysis and RT-qPCR results was 75%, indicating that the sequencing results had relatively high reliability.

## Discussion

AS is an important pathological basis for cardiovascular and cerebrovascular events such as coronary heart disease and stroke. It is a chronic maladaptive and irreversible inflammatory disease caused by the interaction between endothelial dysfunction and subcutaneous lipoprotein accumulation [27]. Currently, exploring effective therapy targets for AS patients continues to be a popular topic in the world. In this study, exosomes were successfully isolated from mouse BMSCs and used to treat AS. It was found that BMSC-EXO treatment significantly reduced TC and TG content and inhibited cell pyroptosis. Then, aortic tissues in the Model group and BMSC-EXO group were sequenced. A total of 3852 DEGs (2168 down-regulated and 1684 up-regulated) were identified. These DEGs were significantly enriched in various biological processes and pathways related to mitochondrial function, metabolism, inflammation, and immune response.

Exosomes are extracellular vesicles released by cells that play a role in intercellular communication by targeting recipient cells to release their contents [28]. Studies have demonstrated that BMSC-EXO play an important role in cardiovascular disease [29, 30]. Zhang et al. found that BMSC-derived exosomal lncRNA Mir9-3hg treatment inhibited cardiomyocyte ferroptosis by modulating the Pum2/PRDX6 axis [31]. Another study showed that BMSC-derived exosomes carrying miR-125b enhanced the viability, inhibited the apoptosis and inflammation of I/R myocardium cells, and restored the cardiac function of I/R rats through regulating SIRT7 [30]. In our study, TEM, NTA and western blot results showed that exosomes were successfully isolated from mouse BMSCs, and then AS was treated with BMSC-EXO. It was evident that BMSC-EXO significantly reduced TC and TG content. Elevated TC is one of the main risk factors for AS [32]. The increased level of TG in the plasma of AS patients is closely related to the occurrence and development of AS, and is an independent risk factor for coronary heart disease [33, 34]. Therefore, we speculated that BMSC-EXO may stabilize AS by reducing TC and TG.

AS is an inflammatory disease associated with endothelial dysfunction, mainly involving three cell types: endothelial cells, macrophages, and smooth muscle cells [35]. Cell death and inflammation are key factors leading to AS [36]. It has been reported that pyroptosis is an important part of the inflammatory response of body and is involved in the occurrence and development of AS [37]. Unlike caspase-3-dependent apoptosis, pyroptosis requires activation of caspase-1 [38, 39]. Caspase-1-dependent pyroptosis requires activation of a typical inflammatory response. As a representative inflammasome, NLRP3 induces pyroptosis by releasing cytokines. The NLRP3 inflammasome activates caspase-1, acts on the precursor molecules of IL-1β and IL-18, and promotes the maturation and secretion of IL-1β and IL-18 to trigger an inflammatory response and further aggravate atherosclerosis [40]. In this study, The expressions of *NLRP3*, *caspase-1* and *GSDMD* were significantly up-regulated in ApoE-/- mice in the model group, however, the expressions of these genes could be decreased after injection of BMSC-EXO. Furthermore, ApoE-/- mice had high concentrations of IL-1β and IL-18, while the concentrations of IL-1β and IL-18 were significantly decreased after injection of BMSC-EXO. IL-1β is an important pro-inflammatory cytokine that plays an important role in AS [41]. A study found that chronic nicotine exposure increased AS by enhancing the production of IL-1β and TNF-α [42]. IL-18 is an inducer of interferon (IFN)-γ with potent activity on inflammatory and vascular cells and is thought to contribute to the pathogenesis of chronic immune-inflammatory processes [43]. Overexpression of IL-18-binding protein has been shown to slow the progression of aortic lesions in apoE KO mice [44]. Combined with our results, it can be inferred that BMSC-EXO may alleviate AS by inhibiting the expression of pyroptosis-related factors (NLRP3, caspase-1 and GSDMD) and attenuating the release of IL-18 and IL-1β.

To further investigate the specific molecular mechanism of BMSC-EXO in AS, aortic tissues in the Model and BMSC-EXO groups were sequenced. A total of 3852 DEGs (2168 down-regulated and 1684 up-regulated) were identified. The up-regulated DEGs were mainly involved in mitochondrial function and metabolism, which may reflect the enhanced energy production and oxidative stress resistance in VSMCs and macrophages. Mitochondria are important organelles for maintaining cellular homeostasis and survival, and their dysfunction has been implicated in the pathogenesis of AS [45]. The citrate cycle (TCA cycle) is a key process for generating ATP and reducing equivalents in mitochondria. The TCA cycle also provides intermediates for biosynthesis and regulates cellular redox balance [46]. Previous studies have shown that BMSC-EXO could improve mitochondrial biogenesis and function in various cell types [47, 48]. Moreover, some of the up-regulated DEGs were associated with thermogenesis, such as UCP1 and PGC1A, which may indicate the activation of brown adipose tissue (BAT) or beige adipocytes. BAT is a specialized tissue for heat generation and energy expenditure, and it has been reported to have anti-atherogenic effects by reducing lipid accumulation and inflammation in the vasculature [49, 50]. Therefore, BMSC-EXO may exert beneficial effects on AS by stimulating thermogenesis and metabolism.

The down-regulated DEGs were mainly involved in extracellular matrix (ECM) components and immune response, which may reflect the reduced plaque vulnerability and inflammation in VSMCs and macrophages. ECM degradation and remodeling are key features of plaque instability and rupture, which can lead to acute cardiovascular events [51]. Previous studies have shown that BMSC-EXO could inhibit ECM degradation and promote ECM synthesis in various cell types [52, 53]. Moreover, some of the down-regulated DEGs were associated with complement and coagulation cascades, malaria, cytokine-cytokine receptor interaction, and chemokine signaling pathway, which are all involved in inflammatory and immune responses. Inflammation and immune activation are crucial drivers of AS progression and complications [54, 55]. Previous studies have shown that BMSC-EXO could modulate inflammatory and immune responses by suppressing pro-inflammatory cytokines and chemokines, inhibiting macrophage polarization and activation, and inducing regulatory T cells [56, 57]. Therefore, BMSC-EXO may exert beneficial effects on AS by stabilizing ECM and attenuating inflammation and immune response.

In addition, we also verified the expression of DEGs by RT-qPCR. Compared with the model group, the expressions of *Ucp1*, *Acsl5* and *Plin1* were significantly up-regulated, and the expressions of *Scd4*, *Apoa1* and *Pltp* were significantly down-regulated in the BMSC-EXO group. *SCD* is the central enzyme for the synthesis of monounsaturated fatty acids in lipid metabolism and is related to tissue metabolism and body adiposity regulation [58]. *SCD4*, a cardiac-specific isoform of *SCD*, is predominantly expressed in the heart and greatly induced in the heart of Scd1−/− mice [59]. *ApoA1* is a major protein component of HDL, and increased ApoA1 gene expression is associated with a reduced risk of AS [60]. *Pltp* is a member of the lipid transfer/lipopolysaccharide-binding protein family [61]. It has been demonstrated that overexpression of *Pltp* induces AS [62], while its deficiency shows the opposite effect [63]. Masson et al. also found in a rabbit model that *Pltp* overexpression increased AS lesions after high-fat diet feeding compared with controls [64]. *Ucp1* is a marker of brown fat cells responsible for cold and diet-induced thermogenesis [65]. Deficiency of *Ucp1* exacerbates dietary obesity-induced endothelial dysfunction, vascular inflammation and AS in mice [66]. Bernal-Mizrachi et al. showed that *Ucp1* expression in aortic smooth muscle cells can lead to hypertension and increase AS [67]. *Acsl5* belongs to the long-chain fatty acyl-CoA synthetase family, and overexpression of Acsl5 promotes TG synthesis in fatty acids [68]. Wang et al. found that the atherogenic diet significantly increased mRNA expression of *Acsl5* compared with the control diet [69]. *Plin1*, a member of the patatin protein family. *Plin1* can regulate the inflammatory polarity of human macrophages and participate in AS plaque development by promoting stable lipid storage [70]. *Plin1* overexpression in macrophages protects ApoE-knockout mice from atheroma development [71]. Combined with the results of this study, we speculate that BMSC-EXO may be involved in AS progression by regulating these DEGs, including *Scd4*, *Apoa1*, *Pltp*, *Ucp1*, *Acsl5* and *Plin1*. However, the detailed effects of these DEGs on AS require further investigation.

Due to the limitations of our study, some questions remain unanswered. First, the specific mechanisms of KEGG pathways in AS are still elusive and require further investigation. Second, whether BMSC-EXO modulate pyroptosis through KEGG pathways is also a question that needs to be addressed in future studies.

## Conclusion

In conclusion, this study identified the mechanism of action of BMSC-EXO to inhibit pyroptosis in AS aortic tissues, improve the reduction of lipid accumulation and inflammatory lesions in AS aortic tissues, and alleviate the progression of AS lesions by a mechanism related to the inhibition of NLRP3/Caspase-1/GSDMD signaling pathway. In addition, BMSC-EXO may reduced AS inflammation through 3852 DEGs involved in various biological processes and pathways related to mitochondrial function, metabolism, inflammation, and immune response. This study provides a theoretical basis for the application of exosomes in the treatment of AS, but more experiments on the feasibility and safety of BMSC-EXO in the treatment of atherosclerosis are needed to improve the findings of this study.

### Electronic supplementary material

Below is the link to the electronic supplementary material.

Supplementary Material 1: Fig. S1. Validation of DEGs. (A) The expression of Acsl5. (B) The expression of Acsl1. (C) The expression of Ucp1. (D) The expression of Scd4. (E) The expression of Apoa1. (F) The expression of Pltp. (G) The expression ofPlin1. (H) The expression of Fabp1. **P < 0.01 vs. Model group; ***P < 0.001 vs. Model group

## Data Availability

The data that support the findings of this study are available on request from the corresponding author.
