# Correction: Bone marrow mesenchymal stem cellsderived exosomes stabilize atherosclerosis through inhibiting pyroptosis

**DOI:** 10.1186/s12872-024-04027-2

**Published:** 2024-07-10

**Authors:** Zhibin Bai, Haolin Hu, Fangfang Hu, Jiajie Ji, Zhenling Ji

**Affiliations:** 1 Center of Interventional Radiology and Vascular Surgery, Department of Radiology, Medical School, Zhongda Hospital, Southeast University, 87 Dingjiaqiao Road, Nanjing, 210009 Jiangsu China; 2 Department of General Surgery, Institute for Minimally Invasive Surgery, Medical School, ZhongDa Hospital, Southeast University, 87 Dingjiaqiao Road, Nanjing, 210009 Jiangsu China

**Correction: BMC Cardiovasc Disord 23, 441 (2023)**

**https://doi.org/10.1186/s12872-023-03453-y**

Following publication of the original article [1], the authors would like to correct some information under ‘RT-qPCR verification’ in the results section and figure caption in supplementary material.

The sentence originally read as:

Compared with the Model group, the expressions of *Ucp1, Acsl5* and *Plin1* were significantly up-regulated in the BMSC-EXO group, while the expressions of *Scd4, Apoa1* and *Pltp* were significantly down-regulated in the BMSC-EXO group (P < 0.05, Figures S1A–F).

It should read as:

Compared with the Model group, the expressions of *Ucp1, Acsl5* and *Acsl1* were significantly up-regulated in the BMSC-EXO group, while the expressions of *Scd4, Apoa1* and *Pltp* were significantly down-regulated in the BMSC-EXO group (P < 0.05, Figures S1A–F).

The figure caption originally read:

Figure S1. Validation of DEGs. (A) The expression of Acsl5. (B) The expression of Acsl1. (C) The expression of Ucp1. (D) The expression of Plin1. (E) The expression of Scd4. (F) The expression of Apoa1. (G) The expression of Pltp. (A) The expression of Fabp1. **P < 0.01 vs. Model group; ***P < 0.001 vs. Model group

The figure caption should read:

Figure S1. Validation of DEGs. (A) The expression of Acsl5. (B) The expression of Acsl1. (C) The expression of Ucp1. (D) The expression of Scd4. (E) The expression of Apoa1. (F) The expression of Pltp. (G) The expression of Plin1. (H) The expression of Fabp1. **P < 0.01 vs. Model group; ***P < 0.001 vs. Model group

The original article has been corrected.
