# Supplementary material for: Bone marrow mesenchymal stem cellsderived exosomes stabilize atherosclerosis through inhibiting pyroptosis
Source: BMC Cardiovasc Disord. 2023 Sep 7;23:441. doi: 10.1186/s12872-023-03453-y (PMC10486039; doi:10.1186/s12872-023-03453-y)
Supplement: Supplementary file 1 — Supplementary Material 1: Fig. S1. Validation of DEGs. (A) The expression of Acsl5. (B) The expression of Acsl1. (C) The expression of Ucp1. (D) The expression of Scd4. (E) The expression of Apoa1. (F) The expression of Pltp. (G) The expression ofPlin1. (H) The expression of Fabp1. **P < 0.01 vs. Model group; ***P < 0.001 vs. Model group [file 12872_2023_3453_MOESM1_ESM.docx]

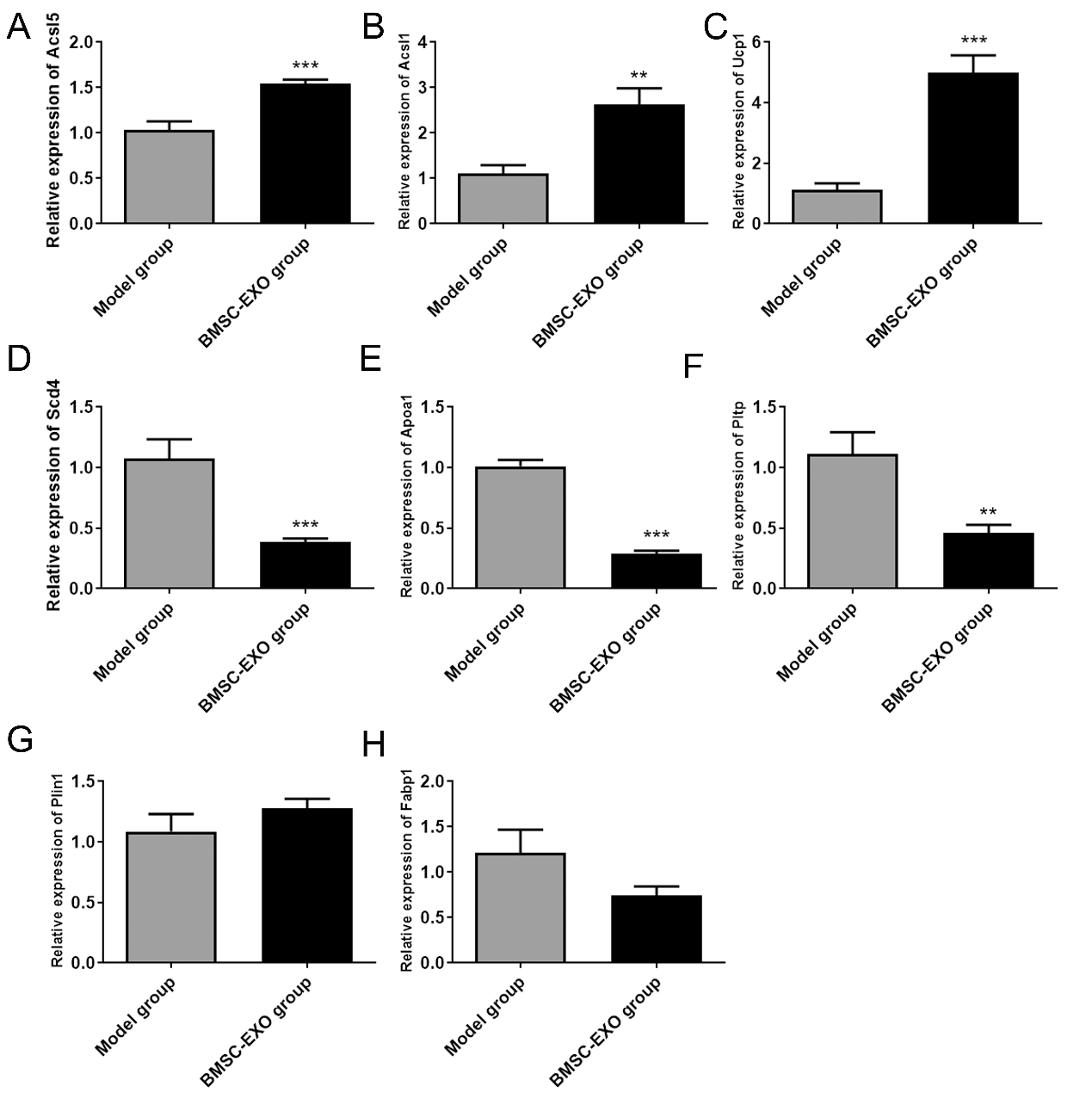


Figure S1. Validation of DEGs. (A) The expression of Acsl5. (B) The expression of Acsl1. (C) The expression of Ucp1. (D) The expression of Plin1. (E) The expression of Scd4. (F) The expression of Apoa1. (G) The expression of Pltp. (A) The expression of Fabp1. ***P* < 0.01 vs. Model group; ****P* < 0.001 vs. Model group.
